# Ensemble Deep Learning and Internet of Things-Based Automated COVID-19 Diagnosis Framework

**DOI:** 10.1155/2022/7377502

**Published:** 2022-02-25

**Authors:** Anita S. Kini, A. Nanda Gopal Reddy, Manjit Kaur, S. Satheesh, Jagendra Singh, Thomas Martinetz, Hammam Alshazly

**Affiliations:** ^1^Manipal Institute of Technology MAHE, Manipal, Karnataka 576104, India; ^2^Department of IT, Mahaveer Institute of Science and Technology, Hyderabad, Telangana 500005, India; ^3^School of Electrical Engineering and Computer Science, Gwangju Institute of Science and Technology, Gwangju 61005, Republic of Korea; ^4^Department of Electronics and Communication Engineering, Malineni Lakshmaiah Women's Engineering College, Guntur, Andhra Pradesh 522017, India; ^5^School of Computer Science Engineering and Technology, Bennett University, Greater Noida-203206, India; ^6^Institute for Neuro- and Bioinformatics, University of Lübeck, Lübeck 23562, Germany; ^7^Faculty of Computers and Information, South Valley University, Qena 83523, Egypt

## Abstract

Coronavirus disease (COVID-19) is a viral infection caused by SARS-CoV-2. The modalities such as computed tomography (CT) have been successfully utilized for the early stage diagnosis of COVID-19 infected patients. Recently, many researchers have utilized deep learning models for the automated screening of COVID-19 suspected cases. An ensemble deep learning and Internet of Things (IoT) based framework is proposed for screening of COVID-19 suspected cases. Three well-known pretrained deep learning models are ensembled. The medical IoT devices are utilized to collect the CT scans, and automated diagnoses are performed on IoT servers. The proposed framework is compared with thirteen competitive models over a four-class dataset. Experimental results reveal that the proposed ensembled deep learning model yielded 98.98% accuracy. Moreover, the model outperforms all competitive models in terms of other performance metrics achieving 98.56% precision, 98.58% recall, 98.75% F-score, and 98.57% AUC. Therefore, the proposed framework can improve the acceleration of COVID-19 diagnosis.

## 1. Introduction

In December 2019, an epidemic of a coronavirus disease (COVID-19) was reported, and due to its rapid spread in the entire world, the World Health Organization (WHO) indicated it as a pandemic [1]. This pandemic has wreaked havoc on the world, affecting all aspects of life including economy, education, society, and environment [2]. The timely diagnosis of infected patients is essential both for infection control and care of the patient [3, 4]. However, an efficient model of COVID-19 diagnosis is yet an immense challenge because of spatial complexity [5, 6]. Advancement in the deep learning-based Internet of Things (IoT) initiated a world of possibilities in healthcare. IoT-based healthcare can overcome the lack of medical specialist issues. Additionally, IoT-based healthcare devices are very helpful for the early diagnosis of various infectious diseases such as COVID-19, HIV, and cancer. The IoT-enabled deep learning model was designed in [7] for automated diagnosis of COVID-19 suspected cases. Recently, many artificial intelligence (AI) approaches have been utilized for automated diagnosis of suspected cases [8–10]. Some of these approaches are extreme learning, convolutional neural network (CNN), generative adversarial networks, and a combination of several handcrafted feature extraction methods [11–14].

A mask-based CNN model (M-CNN) was designed with an almost limited volume of trainable attributes for COVID-19 diagnosis [15]. A joint learning model (JLM) was implemented to achieve efficient COVID-19 diagnosis [16]. The contrastive cross-site training was also implemented by using a redesigned net [16]. A weakly supervised deep learning model (WSDL) was implemented for the diagnosis of COVID-19 suspected cases from CT images. It can reduce the demands of guided labeling of CT images but yet be capable to achieve efficient performance [17]. An iteratively pruned ensemble convolutional neural network (IPCNN) was designed for diagnosing COVID-19 suspected cases. Pretrained ImageNet models were also utilized to train modality-specific features [18]. Three dense CNN models (DeCNN) were utilized, and their respective results were integrated to improve the COVID-19 diagnosis performance [19]. The deep learning-based chest radiograph diagnosis (DLCRD) was proposed for diagnosis of COVID-19 suspected cases [20, 21]. Two 3D ResNets were integrated using a prior-attention approach. The residual learning was improved using the designed prior-attention approach referred to as prior-attention residual learning (PARL) block [22].

The classifier generative adversarial network (i.e., COVIDGAN) was implemented to obtain synthetic chest X-ray images. It has been shown that the obtained synthetic images have improved the accuracy of CNN-based COVID-19 diagnosis models [23]. Adaptive feature selection guided deep forest (AGGDF) was designed for the diagnosis of COVID-19 suspected case [24]. The genetic CNN (GCNN) was implemented. The GCNN was trained from scratch to extract potential characteristics for diagnosing suspected cases [25].

Alshazly et al. [26] adopted various deep network architectures along with a transfer learning strategy for the automated detection of COVID-19 based on CT scans. Extensive experiments were conducted on two CT image datasets, namely, the SARS-CoV-2 CT scan [27] and the COVID-19 CT [28]. Their results indicated superior performance for the adopted models compared with standard ones. In [29], two CNN models were proposed, i.e., CovidDenseNet and CovidResNet, for diagnosis of COVID-19 by considering CT images. The architectures utilized transfer learning to be partly initialized from larger pretrained deep models, which revealed to significantly improve the diagnosis accuracy. The built models can efficiently distinguish between COVID-19, viral pneumonia, and healthy CTs. Experiments were conducted on 4173 CT scans. It was found that CovidResNet and CovidDenseNet have significantly improved the diagnosis performance.

Biswas et al. [30] proposed an effective COVID-19 prediction model based on chest CT images by utilizing transfer learning from three standard deep models, namely, VGG16, ResNet50, and Xception. To improve the overall prediction performance, they proposed ensembled three pretrained models. Experiments carried out on the SARS-CoV-2 CT dataset indicated the effectiveness of their proposed method. A multiobjective differential evolution-based CNN model was presented for classification of COVID-19 infected patients using chest CT scans [31]. Experimental results for binary classification scenarios revealed the superior performance of the proposed model compared with other competitive models under different splits of training and testing datasets. For a comprehensive and systematic review on the different machine learning techniques applied to detect and diagnose COVID-19 from chest radiographic images including X-ray and CT scans, please see [32, 33].

Although the existing deep learning models have achieved significantly better performance for COVID-19 diagnosis, still a majority of the deep learning models suffer from the overfitting problems [34, 35]. Also, deep learning models have millions of parameters that are optimized using stochastic gradient descent algorithms [6, 36]. Thus, the search space contains a large number of local minima that should be avoided. Therefore, in this study, a deep ensemble model is proposed.

The main contributions of this study are as follows.

1. A novel ensemble deep learning and IoT-based frameworks are proposed for screening of suspected cases
2. Three deep learning variants, i.e., ResNet152V2, DenseNet201, and InceptionResNetV2 (IRNV2) are ensembled for automated screening of COVID-19 suspected cases
3. Comparisons are drawn among the proposed and the existing models by using various performance metrics

The rest of the study is organized as follows. In Section 2, preliminaries are discussed. The proposed framework is illustrated in Section 3.  Section 4 presents the comparative analysis.  Section 5 concludes the study.

## 2. Preliminaries

This section presents the background of convolutional neural networks (CNNs). Thereafter, the concept of transfer learning is discussed. Finally, we explain the deep ensemble construction process.

### 2.1. Convolutional Neural Network

**Figure 1:**
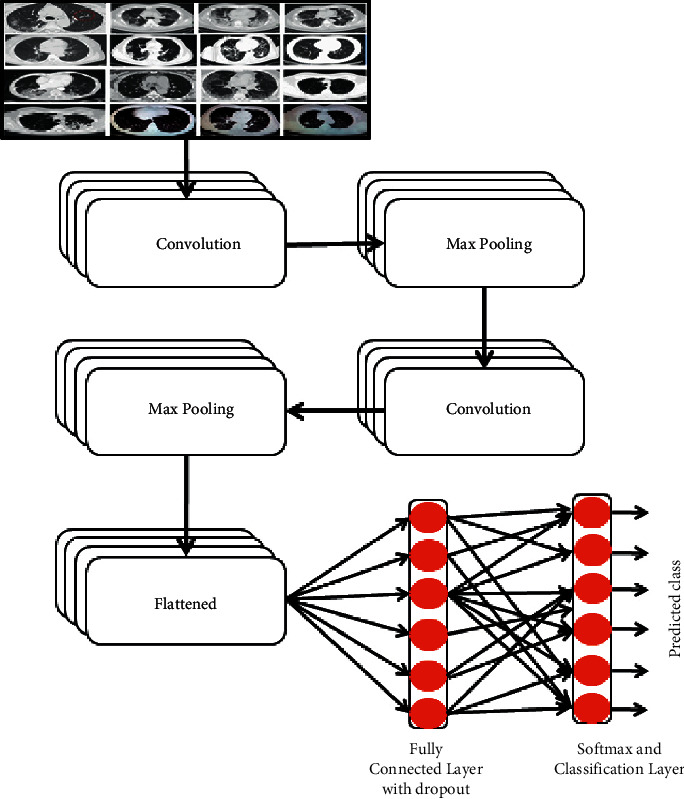
Diagrammatic flow of the CNN model.

The CNN has extensively utilized a supervised learning model motivated by the natural visual attention process of living individuals. The CNN is preferred over the machine learning models as it does not require feature extraction techniques as a preprocessing tool [37, 38]. A CNN is a deep neural network with various convolutional layers as shown in Figure 1. Each convolutional layer contains various filters to apply a convolutional operator on the input image. Pooling is used to subsample the pixels to make the image smaller. Therefore, each layer reduces the attributes to classify the image. Depending upon the number of classes, the sigmoid or softmax activation function is used to evaluate the probability of a given class. However, the CNN requires training with tons of images before performing a logical classification. Also, model building is expensive, both in terms of resources and time [39, 40]. Therefore, transfer learning models are used.

### 2.2. Transfer Learning

**Figure 2:**
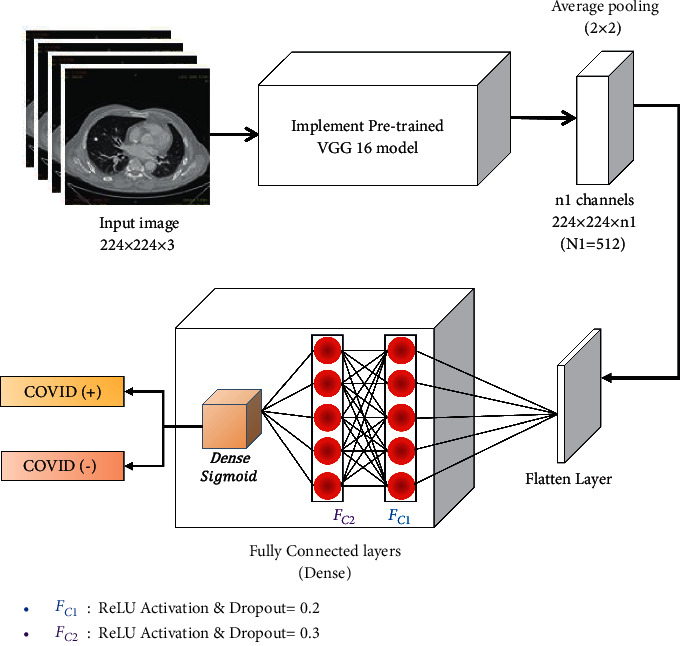
Diagrammatic flow of the VGG16-based transfer learning model for COVID-19 diagnosis.

Given the enormous resources necessary to train deep networks or the large and challenging datasets on which deep networks are trained, transfer learning has become a popular approach in deep learning. Transfer learning is utilized to build a given model by partially utilizing a pretrained model on a different problem. It is preferred especially when we have a lack of data to train deep models from scratch such as the COVID-19 dataset. It can reduce the training time, and also, it does not require setting up a costly processing unit.  Figure 2 shows a VGG16 [41] based transfer learning model for diagnosis of COVID-19 suspected cases. It is found that the pretrained model is utilized to extract the features of chest CT images. Finally, a fully connected layer of the CNN model along with a dropout is used to classify the results [42]. This fine-tuning strategy of deep CNN models has proved to be the most effective approach of transfer learning, which incrementally adapts the pretrained features to the new data [43]. Some recently utilized deep learning models are as ResNet152V2 [44], DenseNet201 [34], and Inception ResNetV2 (IRNV2) [3].

### 2.3. Deep Ensemble

Deep CNNs have millions of parameters that we attempt to optimize through a stochastic gradient descent algorithm or its variants. As a result, the search space contains a large number of local minima, which the optimizer tries to avoid but frequently converges to. Eventhough these networks achieve comparable error rates, they make different mistakes due to the vastness of their search space. As a result, their diversity can be used to exploit by building ensembling techniques [45, 46].

The two common approaches for constructing ensembles are homogeneous and heterogeneous ensembling. In homogeneous ensembles, the stochasticity of the training process is exploited, where we train the same network architecture multiple times with random or different initializations. On contrary, in heterogeneous ensembles, the goal is to train different network architectures to exploit their complementary descriptive powers. Following the training process, the networks can be integrated with various ways such as majority voting, averaging, or concatenating the output of the penultimate layer and subsequently performing classification by an external classifier. In this regard, constructing heterogeneous ensembles of deep CNN models of varying depths has been found to achieve state-of-the-art results in various vision tasks including biometrics [47], scene classification [48], and building robust diagnostic systems [49–51].

**Figure 3:**
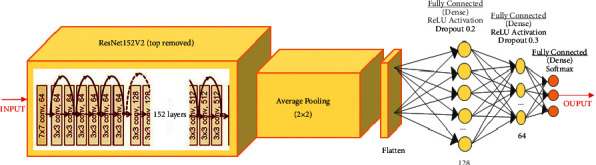
Diagrammatic flow of the ResNet152V2 model.

**Figure 4:**
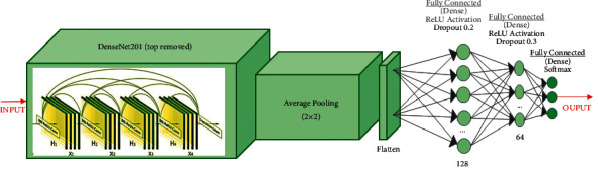
Diagrammatic flow of the DenseNet201 model.

**Figure 5:**
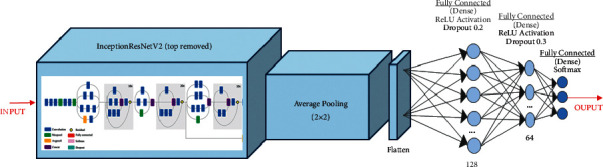
Diagrammatic flow of the IRNV2 model.

Figures 3–5 show ResNet152V2, DenseNet201, and IRNV2-based pretrained models. The neurons for an initial dense layer are set to be 64. The fine-tuned transfer learning models are utilized to extract the features. The softmax activation function is used. All pretrained models are built for 250 epochs. For fine-tuning, the Adam optimizer [52] is utilized. Early stopping is implemented to achieve regularization which can overcome the overfitting issue.

## 3. Proposed Model

**Figure 6:**
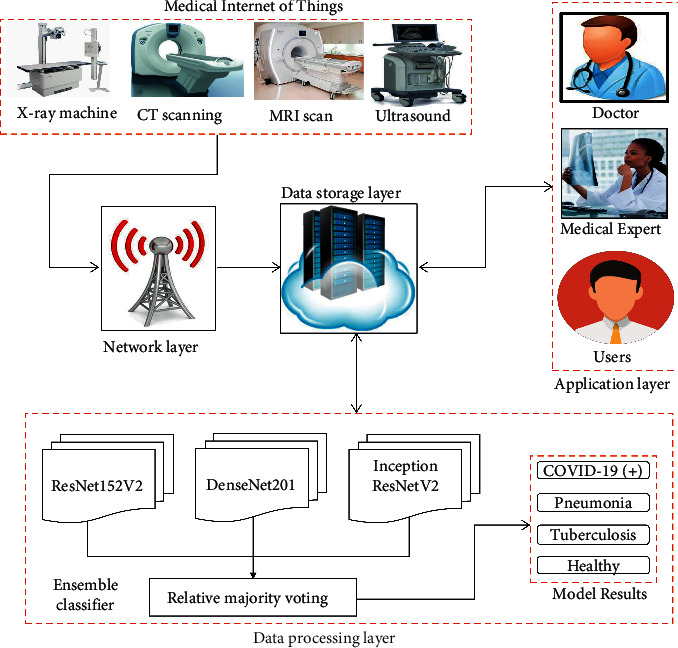
Diagrammatic flow of the proposed IoT-enabled ensemble deep learning model for automated screening of COVID-19 suspected cases.

The proposed IoT-enabled ensemble deep learning model is shown in Figure 6. Initially, medical IoT devices obtain the required scan of a patient at a local hospital or medical center. The collected scans are then transferred to the storage layer of IoT framework using some communication media. Thereafter, processing of the obtained scan is done with the help of the ensemble deep learning model. The returned outcomes are then stored on the data storage layer. Different types of IoT users such as medical experts, doctors, and patients can access their results from the storage layer.

**Figure 7:**
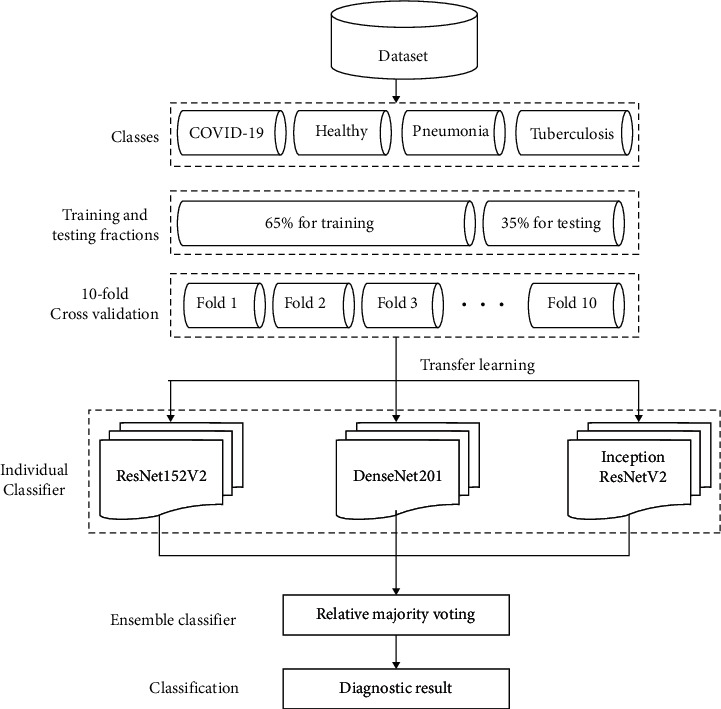
The proposed ensemble deep learning model.

The proposed ensemble deep learning model is shown in Figure 7. It clearly shows that initially, we will train the pretrained models individually. Thereafter, majority voting is implemented to obtain the final ensembled framework for automated screening of COVID-19 suspected cases. The remaining section discusses the step-by-step ensemble model.

(1) Initially, a four-class chest CT dataset is obtained
(2) Divide the dataset into training and testing fractions, i.e., 65% and 35%, respectively. Ten-fold (10-fold) cross-validation is then applied to obtain 10 uniform sets.
(3) Mathematically, each set is defined as
  (1) $$\begin{array}{c} \left[C_{tr}C_{ts}\right]=T_{f}\left(D_{S}\right). \end{array}$$
Here, *C*_*tr*_ represents the training set of CT scans, *C*_*ts*_ shows the testing set, *T*_*f*_ is the 10-fold cross-validation, and *D*_*S*_ is the collected four-class CT scan dataset.
(4) The deep learning models, i.e., ResNet152V2, DenseNet201, and IRNV2 are applied on the testing dataset (*C*_*ts*_) as
  (2) $$\begin{array}{c} R_{S}=T_{L}\left(R,S\right),\\ D_{S}=T_{L}\left(D,S\right),\\ I_{S}=T_{L}\left(I,S\right). \end{array}$$
Here, *R*_*S*_, *D*_*S*_, and *I*_*S*_ show the softmax functions of ResNet152V2, DenseNet201, and IRNV2, respectively, *T*_*L*_ represents the deep transfer leaning model, *R*, *D*, and *I* demonstrate the ResNet152V2, DenseNet201, and IRNV2, respectively, and *S* shows the softmax function.
(5) The trained individual deep transfer learning models can be defined as
  (3) $$\begin{array}{c} R_{S}=M_{B}\left(R_{S},C_{tr}\right),\\ D_{S}=M_{B}\left(D_{S},C_{tr}\right),\\ I_{S}=M_{B}\left(I_{S},C_{tr}\right). \end{array}$$
Here, *M*_*B*_ defines the model building process.
(6) Finally, ensembling is achieved by using the majority voting as
  (4) $$\begin{array}{c} \begin{array}{c} E_{C}=E_{M}\left(R_{S},D_{S},I_{S}\right) \end{array}. \end{array}$$
Here, *E*_*C*_ is the trained ensemble diagnostic model. *E*_*M*_ defines the majority voting ensemble model.

## 4. Performance Analysis

The proposed framework is realized on online MATLAB 2020b software with the support of a deep learning toolbox. To give a comprehensive analysis and comparison, we have implemented numerous COVID-19 diagnostic models such as the JLM [16], WSDL [17], IPCNN [18], DeCNN [19], DLCRD [20], PARL [22], AGGDF [24], GCNN [25], GoogLeNet [53], ResNet152V2 [44], DenseNet201 [34], and IRNV2 [3].

The chest CT dataset is collected from various sources [54–58]. It contains 2839, 2632, 3193, and 3482 CT scans images of COVID-19 (+), tuberculosis, pneumonia, and healthy persons, respectively.

**Figure 8:**
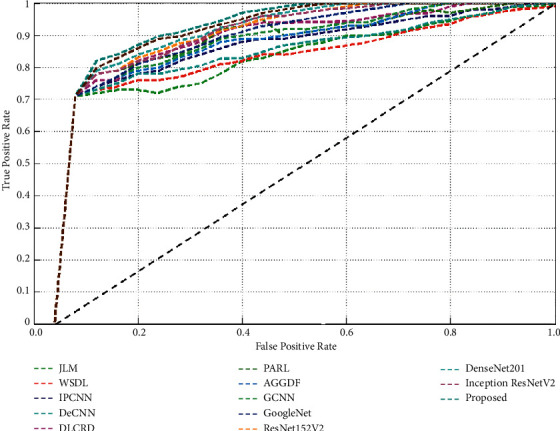
ROC analysis of the proposed IoT-enabled ensembled deep learning framework.

The receiver operating characteristic curve (ROC) analysis of the proposed IoT-enabled ensemble deep learning framework is shown in Figure 8. The proposed framework achieves better area under curve (AUC) values with an average AUC value of 98.57% which is remarkably better than the competitive models. Therefore, the proposed framework can be efficiently applied for the early diagnosis of COVID-19 suspected cases.

Tables 1 and 2 provide the training and testing analyses of the proposed IoT-enabled ensembled deep learning framework. The proposed model obtains average training results in terms of accuracy, *F*-measure, AUC, recall, and precision of 99.12%, 98.91%, 98.79%, 99.28%, and 99.08%, respectively. Whereas on testing data, the model achieves remarkably good results in terms of accuracy, *F*-measure, AUC, recall, and precision of 98.97%, 98.75%, 98.57%, 98.58%, and 98.56%, respectively. Overall analysis indicates that the proposed approach achieves better results than the existing models in terms of accuracy, *F*-measure, AUC, recall, and precision by 1.19%, 0.98%, 0.95%, 1.04%, and 1.02%, respectively.

The experimental analysis demonstrates that the proposed model has better performance than the JLM [16], WSDL [17], IPCNN [18], DeCNN [19], DLCRD [20], PARL [22], AGGDF [24], GCNN [25], GoogLeNet [53], ResNet152V2 [44], DenseNet201 [34], and IRNV2 [3] based COVID-19 diagnostic models. Additionally, the proposed model is designed for IoT networks and therefore can provide rapid testing results even for remote users. The proposed framework can also test tuberculosis and pneumonia patients at the same time. Since the proposed model is also IoT-enabled, therefore, it initially diagnoses the type of infection and provides results. If the given patient is infected from COVID-19, then the patient's sample will be further processed for the verification of actual COVID types such as delta variant and omicron variant. Thus, the proposed framework can provide better performance and can be used for automated diagnosis of COVID-19 suspected cases.

## 5. Conclusion

A novel IoT-enabled deep learning framework for automated COVID-19 diagnosis framework has been proposed. Three well-known fine-tuned transfer learning models were utilized to design an ensemble model. The majority voting classifiers were utilized to ensemble the outcomes of deep transfer learning models. The proposed model was tested on a four-class (i.e., COVID-19, pneumonia, tuberculosis, and healthy cases) chest CT dataset. Extensive experiments have been conducted by considering the proposed framework and several existing models using various performance evaluation metrics. It has been observed that the proposed framework outperforms the competitive models in terms of AUC, accuracy, recall, *F*-measure, and precision by 1.19%, 0.98%, 0.95%, 1.04%, and 1.02%, respectively. Because the proposed model is also IoT-enabled, it can diagnose the type of infection and provide results right away. If a patient has been infected with COVID-19, the patient's sample will be analysed further to determine the COVID type such as delta and omicron variations. Therefore, the proposed framework can improve the acceleration of COVID-19 diagnosis.

## Figures and Tables

**Table 1:** Training analysis (%) of the proposed IoT-enabled deep ensemble model for automated diagnosis of COVID-19 suspected cases on the four-class chest CT dataset when training to testing ratio is 65 : 35.

Model	Accuracy	*F*-measure	AUC	Recall	Precision
JLM [16]	97.88	98.27	98.07	98.97	97.83
WSDL [17]	98.11	98.56	98.33	99.14	97.89
IPCNN [18]	97.93	97.63	97.78	98.54	97.38
DeCNN [19]	98.45	97.53	97.99	98.89	97.82
DLCRD [20]	98.43	97.64	98.03	98.94	97.73
PARL [22]	98.67	97.44	98.05	98.76	98.12
AGGDF [24]	98.17	97.65	97.91	98.72	97.65
GCNN [25]	98.68	97.58	98.13	98.97	98.21
GoogLeNet [53]	98.16	98.35	98.25	99.08	97.62
ResNet152V2 [44]	98.55	98.33	98.44	99.27	97.85
DenseNet201 [34]	98.57	98.18	98.34	99.09	97.83
IRNV2 [3]	98.18	97.48	97.83	98.67	97.56
Proposed	99.12	98.91	98.79	99.28	99.08

**Table 2:** Testing analysis (%) of the proposed IoT-enabled deep ensemble model for automated diagnosis of COVID-19 suspected cases on the four-class chest CT dataset when training to testing ratio is 65 : 35.

Model	Accuracy	*F*-measure	AUC	Recall	Precision
JLM [16]	97.46	97.84	97.65	97.98	98.08
WSDL [17]	96.96	97.17	97.06	97.46	97.88
IPCNN [18]	97.95	97.55	97.75	98.03	98.03
DeCNN [19]	97.36	97.36	97.18	97.27	98.11
DLCRD [20]	98.13	98.12	98.14	98.19	97.63
PARL [22]	97.11	97.52	97.31	97.78	97.93
AGGDF [24]	97.73	97.44	97.58	97.75	97.47
GCNN [25]	97.46	97.75	97.65	97.92	97.99
GoogLeNet [53]	97.77	97.33	97.55	97.69	97.94
ResNet152V2 [44]	96.96	97.89	97.42	97.74	97.67
DenseNet201 [34]	97.44	97.59	97.51	97.87	98.11
IRNV2 [3]	98.06	97.98	98.02	98.27	98.36
Proposed	98.97	98.75	98.57	98.58	98.56

## Data Availability

The data used to support the findings of this study are available from the corresponding author upon request.
